# Assessment of providers' referral decisions in Rural Burkina Faso: a retrospective analysis of medical records

**DOI:** 10.1186/1472-6963-12-54

**Published:** 2012-03-08

**Authors:** Tegawende Pierre Ilboudo, Yiing-Jenq Chou, Nicole Huang

**Affiliations:** 1 Institute of Public Health, School of Medicine, National Yang Ming University, Section 2, Li-Nong Street, Taipei 112, Taiwan; 2 Service de Lutte contre la Maladie et Protection des Groups Spécifiques, Direction Régionale de la santé du Centre-Est, Ministère de la Santé, BP 7009, Ouaga, Burkina Faso; 3 Institute of Hospital and Health Care Administration, School of Medicine, National Yang Ming University, Room 201, The Medical Building II, No.155, Section 2, Li-Nong Street, Taipei 112, Taiwan

**Keywords:** Diagnostic errors, Compliance, Healthcare quality improvement, Patient safety, Primary care

## Abstract

**Background:**

A well-functioning referral system is fundamental to primary health care delivery. Understanding the providers' referral decision-making process becomes critical. This study's aim was to assess the correctness of diagnoses and appropriateness of the providers' referral decisions from health centers (HCs) to district hospitals (DHs) among patients with severe malaria and pneumonia.

**Methods:**

A record review of twelve months of consultations was conducted covering eight randomly selected HCs to identify severe malaria (SM) cases among children under five and pneumonia cases among adults. The correctness of the diagnosis and appropriateness of providers' referral decisions were determined using the National Clinical Guidebook as a 'gold standard'.

**Results:**

Among the 457 SM cases affecting children under five, only 66 cases (14.4%) were correctly diagnosed and of those 66 correctly diagnosed cases, 40 cases (60.6%) received an appropriate referral decision from their providers. Within these 66 correctly diagnosed SM cases, only 60.6% were appropriately referred. Among the adult pneumonia cases, 5.9% (79/1331) of the diagnoses were correctly diagnosed; however, the appropriateness rate of the provider's referral decision was 98.7% (78/79). There was only one case that should not have been referred but was referred.

**Conclusions:**

The adherence to the National Guidelines among the health center providers when making a diagnosis was low for both severe malaria cases and pneumonia cases. The appropriateness of the referral decisions was particularly poor for children with severe malaria. Health center providers need to be better trained in the diagnostic process and in disease management in order to improve the performance of the referral system in rural Burkina Faso.

## Background

A well-functioning referral system is fundamental to primary health care (PHC) delivery. Through referrals, primary care facilities save lives and provide prompt responses to emergency situations; they do this by helping people obtain access to higher levels of care, particularly at the district level [1]. Evidence has shown that intervention aimed at improving the referral system should concentrate on improving referral appropriateness rather than controlling the referral rate [2]. Assessing and understanding the providers' referral decision-making process become critical. Findings from developed countries have shown that a referral decision is determined by a complex mix of patient, provider and organization characteristics [3-6]. In the developing world, very few published studies are available. Previous studies have mainly focused on the appropriateness of hospital admissions [7]. However, analyzing hospital admissions to assess referral appropriateness is very likely to omit referrals that did not attend the hospital. A more straightforward assessment of referral appropriateness is to analyze primary care providers' decisions directly. Despite the obvious importance of referral appropriateness assessment, in sub-Saharan Africa, to our knowledge, very few published studies have assessed the appropriateness of primary care providers' referral decisions [8].

In Burkina Faso, nurses have been authorized to diagnose and prescribe because there is a shortage of physicians (1 physician for 20,000 inhabitants). They substitute for general practitioners in rural areas, particularly in health centers (HCs). To improve the standard of care at HCs, a widely accepted guide to diagnosis and treatment (The National Guidebook for Diagnosis and Treatment) is used as a reference for diagnosis, treatment and referral decision making. Previous studies have applied the Guidebook when assessing the quality of physical examinations, the diagnostic process and drug prescription [9-13]. However, up to the present, no study has yet evaluated the appropriateness of the provider's referral decisions. Assessing the appropriateness of the health center providers' decisions regarding referral would help to fill this gap in the literature and provide evidence as to what actions could be undertaken to improve the performance of providers and the referral system. A referral decision process involves two levels. The first level is whether patient is diagnosed correctly, while the second level is, if diagnosed correctly, whether the patient is referred when they need to be referred. Based on the above, this study aimed to assess how coherent these HC staff diagnosed or made a referral decision according the clinical guidebook for two indicator conditions, severe malaria in children and pneumonia in adults. The guidebook served as a standard to evaluate correctness and appropriateness of provider's diagnoses and referral decisions in HCs.

## Methods

### The study setting

The Ouargaye district is one of 63 medical districts in Burkina Faso. It is located in the southeastern part of the country close to the Togo-Burkina-Ghana border and is about 250 km from Ouagadougou, the capital city. The total surface area of the district is 5,656 km^2 ^and the population has been estimated to be 248,394 inhabitants (2008). The district consists of one district hospital (DH) and 25 HCs, which are managed by 150 health workers. The physicians to population and nurse to population ratios are 0.03 and 0.32 per 1000 persons. Each HC is a first level health care facility and serves as an interface between the health system and community. It offers an essential package of health services (curative care, preventive care and promotional care) as well as management activities (epidemiological surveillance and reports together with drug, logistic, and financial management). In the Ouargaye district, geographic accessibility to the health services is low, with about 40% of the population living 10 km or more from any health facility. Health service utilization is also relatively low at 0.6 visits for curative care per person per year. In 2007, 148,145 visits for curative purpose were received at the HCs and 2,789 patient referrals to the DH occurred (referral rate = 2%). Infectious diseases represent 80% of the consultations for curative purpose. Malaria (56%) and Acute Low Respiratory Infections (ALRI, 16%) are the leading reasons of consultation. As pneumonia in adults represents 32% of the ALRI cases and SM is the leading cause of mortality, especially in children under five, these two conditions were chosen to be the indicator conditions in the present study [14].

### Sample and data

Based on the previous literature, we excluded six HCs from the sampling frame as they are located more than four hours from the DH [15]. A stratified sampling strategy was applied to the 19 remaining HCs. The 19 HCs were stratified into four categories according to their travel time from the DH (cut point = 1 hour), and the catchment population size (cut point = 10,000 inhabitants). The purpose of this was to control for travel time to the district hospital because, intuitively, travel time may influence referral compliance. Furthermore, as the HCs vary significantly in their served population, from the smallest at 5,421 inhabitants to the largest at 16,039 inhabitants, it is likely that practice patterns of providers at the HCs serving a larger population may differ from those serving a smaller population. To stratify by catchment population size would allow us to include HCs serving different population sizes. Next, two HCs were randomly selected in each of the four subgroups using SPSS 15.0 software. The eight selected HCs employed 26 health workers and covered a population of 74,748 inhabitants. A pair of nurses at each HC was selected for the record review. All nurse field researchers received two-day training and underwent a practical exercise at a non-selected HC. A surveyor guideline was given to help the fieldwork run smoothly. All the field researchers filled in a confidentiality form.

The study was conducted from July 12 to August 31, 2008. The records of all consultations in the previous 12 months (from July 1, 2007 to June 30, 2008) were reviewed to identify SM cases among children under five and pneumonia cases among adults (15 years and above). From the 45,356 consultations reviewed, 457 SM cases among children and 1,331 pneumonia cases among adults were identified. Information related to patient identity, patient residence, symptoms/physical signs, diagnosis, provider's characteristics, and provider's referral decision, were also collected.

### Dependent variables

The National Guidebook for Diagnosis and Treatment was developed in the year 2003 by the Ministry of Health of Burkina Faso. The training of HC staff in the National Guidelines is a 5-day training program. Each session enrols 20-30 providers. Every HC staff is required to attend the training program. The training contents include diagnosis of prevalent diseases/conditions, construction of a decision algorithm, use of the clinical guidelines, identification of the "gate symptom/sign," prescription of corresponding treatment, and making a referral according to the guidelines. One weakness of the training is that it does not include a practice session at a district hospital. However, the trainees receive post-training supervision from instructors and quarterly supervision from the district management team. At the HC level, providers are advised to use this guidebook to diagnose and manage cases. In addition, since the guidebook has incorporated all the directives of the specific health programs, it has become the only standard for curative care management at the HC level in Burkina Faso. Hence, the Guidebook was used in this study to assess whether the diagnosis was correct and whether the providers' decisions were appropriate.

A case was defined as having a correct diagnosis if this case had recorded symptom(s) or sign(s) that were consistent with the symptom(s) or sign(s) for that diagnosis in the Guidebook. A case was defined as having an incorrect diagnosis if the case had symptom(s) or sign(s) that were different from those indicated for that diagnosis in the Guidebook, including the condition when a diagnostic procedure (like a spinal fluid check) was not performed. All symptoms or signs had to be consistent with the Guidebook.

An 'appropriate referral decision' was defined as a provider's referral decision which was consistent with the recommendations in the Guidebook, given that there has been a correct diagnosis. When the diagnosis was incorrect, the case was not submitted to the appropriateness assessment. An 'over-referral case' was defined as a referral that 'should not' have been referred according to the Guidebook and an 'under-referral case' was defined as a non-referred case that 'should' have been referred.

### Statistical analyses

The appropriateness rate was calculated as the percentage of appropriate decisions. A χ^2 ^test and/or Fisher's exact test were performed as appropriate. Cluster-adjusted χ^2 ^tests were also conducted. The results remained robust. Dataset collected was entered in an Excel 2007 matrix and exported to the SPSS 15.0 software package for analysis. To assess the statistical significance, the alpha level was set at 0.05 for all analyses. This study has fully complied with the Helsinki Declaration and received approval from the *Ministere de la Sante *of Burkina Faso. The reference number is 2008-0678/MS/SG/DRS-CE.

## Results

### Severe malaria among children under five

Table 1 shows the characteristics of the 457 cases of SM among children under five. For pediatric SM, the highest proportion of cases occurred in the age group < 12 months (29.5%). Of these children with SM, 56.9% were boys and 43.1% were girls. A physical sign was present in 76.1% of the cases. The main symptoms were a hot body (95.4%), vomiting (32.6%), other digestive complaints (10.9%), and coughing (6.8%), whereas the main physical signs were fever (95.4%), neurological signs (65.4%), anemia/paleness (9.2%), and dyspnea (4.6%). Only 12% of the SM cases were referred. 88% of the cases remained to be managed at the HC. In terms of provider characteristics, almost all the cases (94.3%) were received by male providers. A majority of the cases (73.5%) were received by providers aged < 30 years old, and 82.3% of the cases were received by nurses.

**Table 1:** Characteristics of the 457 severe pediatric malaria cases and the 1331 adult pneumonia cases from the Ouargaye District, (July 2007-June 2008).

Variables	Severe malaria		Pneumonia	
	
	N (457)	%	N (1331)	%
**Patient characteristics**				

Age (months)				

0-11	135	29.5		

12-23	118	25.8		

24-35	106	23.2		

≥ 36	98	21.4		

Age (years)				

15-29			1117	83.9

30-44			113	8.5

45-59			47	3.5

≥ 60			54	4.1

Gender				

Male	260	56.9	609	45.8

Female	197	43.1	722	54.2

Physical signs				

Present	348	76.1	395	29.7

Absent	109	23.9	936	70.3

Referred				

Yes	55	12.0	4	0.3

No	402	88.0	1327	99.7

**Provider characteristics **				

Age of care provider (years)				

≤ 30	336	73.5	701	52.7

> 30	121	26.5	630	47.3

Gender of care provider				

Male	431	94.3	1268	95.3

Female	26	5.7	63	4.7

Qualification of care provider				

Nurses	376	82.3	918	69.0

Other providers*	81	17.7	413	31.0

*Auxiliary Midwives, Itinerant Health Workers

Table 2 shows that 14.4% (66/457) of the cases were diagnosed correctly by the providers according to the Guidebook. Table 3 indicates that among the 66 SM cases with a correct diagnosis, the appropriateness rate of the provider's referral decision was 60.6% (40/66). More specifically, 11 of these 66 correctly diagnosed SM cases should have been referred to the DH, but only 8 of them were actually referred. In total, 3 cases (4.5%) were potentially inappropriately kept at the HC (under-referral). On the other hand, 55 of the 66 correctly diagnosed cases should have remained at the HC for treatment, but only 32 of them were actually kept at the HC. Thus 23 cases (34.9%) were potentially inappropriately referred to the DH (over-referral).

**Table 2:** Correctness of the diagnosis by condition.

	Correct Diagnosis		Incorrect Diagnosis		Total	
	**N**	**%**	**N**	**%**	**N**	**%**

Severe malaria	66	14.4	391	85.6	457	100.0

Pneumonia	79	5.9	1252	94.1	1331	100.0

**Table 3:** Appropriateness of the provider's referral decision regarding for severe malaria among children under five who had a correct diagnosis (n = 66)

	Referred		Not Referred		Total	
	**N**	**%**	**N**	**%**	**N**	**%**

Were referred	8	12.1	3	4.5	11	16.7

Were not referred	23	34.9	32	48.5	55	83.3

Total	31	47.0	35	53.0	66	100.0

Clustered-adjusted χ^2 ^= 2.94, *p *= 0.087

### Pneumonia among adults

Among the 1331 pneumonia cases, 83.9% of the patients were aged between 15 and 29 years and 45.8% were male. A physical sign was present in only 29.7% of the cases. The main symptoms were coughing (96.6%), chest pain (40.0%), a hot body (34.9%), and headaches (14.4%), whereas the main physical signs were dyspnea (28.5%) and rales (3.5%). As a general symptom, fever was present in 34.9% of the cases. The referral rate (4/1331) was 0.30%. Overall, 95.3% of the cases were received by male providers. Slightly more than half of the adult pneumonia cases were received by providers < 30 years old. Furthermore, 69.0% of the cases were received by nurses whereas 31.0% were received by auxiliary midwives and itinerant health workers.

Table 2 shows that 5.9% of the cases (79/1331) were diagnosed correctly by the providers according to the Guidebook, which was substantially lower than that observed for the SM cases in children under five. Table 4 indicates among the pneumonia cases with a correct diagnosis, the appropriateness rate of the provider's referral decision was 98.7% (78/79). According to the Guidebook, none of these 79 correctly diagnosed pneumonia cases should have been referred to the DH. However, one case was actually referred and thus was an over-referral.

**Table 4:** Appropriateness of the provider's referral decision regarding pneumonia cases among adults who had correct diagnosis (n = 79)

	Referred		Not Referred		Total	
	**N**	**%**	**N**	**%**	**N**	**%**

Were referred	0	0.0	0	100.0	0	0

Were not referred	1	1.3	78	98.7	79	100.0

Total	1	1.3	78	98.7	79	100.0

Clustered-adjusted χ^2 ^= 77.03, *p *< 0.001

## Discussion

This study has two important findings. Firstly, the diagnostic ability of health center providers in rural Burkina Faso was poor for either SM among children under five or pneumonia among adults. According to the Guidebook, only 1 in 7 SM cases and 1 in 17 pneumonia cases were diagnosed correctly. These results suggest that providers do not adhere to the Guidebook as recommended. Based on the written observations of our nurse field researchers, the chief reasons for an incorrect SM diagnosis were the lack of a spinal fluid check when there were neurological signs, and a lack of information about some negative signs like non-oscillating fever and non-dissociated pulse. On the other hand, the main reasons for an incorrect pneumonia diagnosis were a lack of information about how long the cough had lasted, the presence of hemoptysis, abnormal respiratory signs, and the lack of information about some negative signs such as an absence of multiple episodes of wheezing or exercise-induced dyspnea.

Two plausible explanations can also be put forward for the incorrectness of diagnoses. These are either the staff did not have sufficient skills to conduct a good physical examination or the staff members were reluctant to use the Guidebook. Previous studies have also shown that the large clinical variability found with pediatric SM can sometimes cause diagnostic errors even in teaching hospitals [16,17]. In practice, in a setting where malaria is predominant and endemic, over-diagnosis is a common concern [18]. Therefore, enforcing the use of the Guidelines, when used in combination with the Rapid Diagnostic Test (RDT) of malaria at health centers, ought to help to reduce diagnostic errors and prevent the inappropriate use of malaria drugs.

When pneumonia is considered, even if this condition has relatively more specific symptoms and signs (cough and rales) than malaria, making an accurate diagnosis using only a clinical examination is not easy even for physicians. Therefore, as nurses are the main providers at health centers in rural Burkina Faso, more training and extensive practice are needed to help nurses to obtain better diagnostic capability. In addition to their level of knowledge and skill, the motivation of nurses at health centers needs to be taken into consideration; nurses may have a good level of knowledge but perhaps this knowledge is not being applied. Furthermore, a previous study in Niger has revealed that a '*fear loss of power and prestige' *was a real concern among nurses [19]. As a result, staff may be reluctant to use the Guidebook, particularly when the client is present. In addition, the providers' perception of the validity of clinical guidelines and their field experience (an effect of 'mind-lines') may influence their likelihood of complying with the guidelines [18]. Our findings concur with previous studies that there is relatively poor diagnostic quality and guideline adherence among medical staff at rural health centers [9-12,20].

The other important finding of this study is that the appropriateness of the provider's referral decision at health centers varied with the medical condition. Whereas the appropriateness rate of the provider's referral decision for adult pneumonia cases was 98.7%, almost 40% of the SM cases with an appropriate diagnosis were referred inappropriately. Despite differences in study population and assessment criteria, our figures are within the range of inappropriateness rates observed in previous studies (44%-74%) [7,21].

The high over-referral rate (34.9%) and low under-referral rate (4.5%) observed in children may reflect a common myth that excessive referral is less harmful to patients than failure to refer in terms of treatment outcome. However, over-referral may also lead to unnecessary costs and adverse health concerns. Numerous studies in children under five with acute conditions have found that there is a referral compliance rate of less than 50% regardless of the referral appropriateness [8,22,23]. Geographic barriers and/or extra financial burden may hinder these rural residents from complying with the referral. Therefore, we may assume that at least half of the 23 children inappropriately referred to the DH did not comply with the referral, and most of them might die. In fact, a previous study done in Burkina Faso found that the case fatality rate for SM in a hospital setting was 14% [16]. Many of these adverse events can have been avoided if they were treated early and appropriately at health centers.

This study has several limitations. Firstly, the use of the National Guidebook as a "gold standard" for appropriateness of the referral decisions may be a concern. Although this assessment has the advantage of using a widely accepted standard, the validity and reliability of the clinical guidelines may need to be further assessed. In addition, such an explicit assessment method is likely to be stricter than an implicit method (expert review). Further studies using more comprehensive appropriateness indicators may help in this regard. Secondly, as only two indicator conditions were investigated in this study, the results may not be generalizable to all other medical conditions. Thirdly, data quality and the completeness of the medical records at the HCs may be a concern as the analyses were limited to the information recorded in the medical records. Poor record keeping with respect to the symptoms recorded or any missing information may lead to an under-estimation of the diagnostic correctness and the referral appropriateness. Fortunately, while data incompleteness may still occur in medical records, this concern does not seem to be pronounced for these two indicator conditions at the health centres under this study as the district has a dynamic District Management Team. This team conducts routine data quality assessments at the HCs. In addition, none of the records had the patient's age missing, which is a sign of good record keeping. Fourthly, due to the retrospective design of the study, information on the staff's knowledge level or their actual use of the guidelines when they attending patients is not available. Thus this study is unable to disentangle the reasons behind the incorrectness/inappropriateness of the provider's diagnosis/decision. Future research using a prospective design may help in this regard. Finally, by excluding the very distant HCs, the generalizability of the results may have been compromised. These distant HCs tend to have fewer interactions with the District Management Board and the supervision team of the DH. Moreover, the distance between the distant HC and the referral hospital easily lead to different decision making between the providers at distant and non-distant health centers. On average, these distant health centers have a lower referral rate (1.5%) and a lower average referral feedback rate (4.4%) than the non-distant health centers (2.0% and 27.2%, respectively). Follow-up studies targeted specifically these distant health centers should help to advance our knowledge in this regard.

## Conclusions

In summary, in a typical rural district of Burkina Faso, we found that a substantial numbers of adult pneumonia were incorrectly diagnosed; nonetheless, those who were correctly diagnosed were appropriately referred. In these circumstances, interventions that will help to improve the providers' diagnostic capability are crucial to the management of pneumonia care. However, the findings were not the same for severe malaria among children under five. The results indicate that, even if the provider is able to correctly diagnose a severe malaria case, they seem to be incapable of making an appropriate referral decision with respect to the child. Over-referral was common for children. To assure the quality management of severe malaria among children will require strong efforts to enhance the providers' diagnostic, treatment and referral capacities. Future research should explore the factors influencing providers' referral decisions with respect to pediatric patients and the findings should help to overcome this problem. Furthermore, the findings support the argument that the development and dissemination of local consensus guidelines alone are not sufficient to improve the referral system [24]. Training and supervision of the providers are also required. Therefore, providers need to be better trained so that they adopt evidence-based guidelines during the diagnostic process and disease management, particularly among children under five. At the same time, a solid evaluation of the validity and reliability of the guidelines are also crucial in order to convince the providers of their worth, which, in turn, will help to improve the performance of the referral system.

## Competing interests

The authors declare that they have no competing interests.

## Authors' contributions

TPI conceived of the study, carried out the data collection and analyses, and drafted the manuscript. YJC participated in the design of the study, performed the data analyses, and assisted in data interpretation and the drafting of the manuscript. NH supervised the study, coordinated analyses, performed data analyses, and helped the drafting of the manuscript. All authors read and approved the final manuscript.

## Pre-publication history

The pre-publication history for this paper can be accessed here:

http://www.biomedcentral.com/1472-6963/12/54/prepub
